# Prevalence, risk factors, psychological effects of children and adolescents with lower urinary tract symptoms: a large population-based study

**DOI:** 10.3389/fped.2024.1455171

**Published:** 2024-08-21

**Authors:** Xingchen Liu, Zhan Wang, Zhaokai Zhou, Shuai Yang, Jing Yang, Yibo Wen, Yanping Zhang, Lei Lv, Jinhua Hu, Qingwei Wang, Wei Lu, Jian Guo Wen

**Affiliations:** ^1^ Paediatric Urodynamic Centre and Department of Urology, The First Affiliated Hospital of Zhengzhou University, Zhengzhou, Henan, China; ^2^ Henan Joint International Paediatric Urodynamic Laboratory, The First Affiliated Hospital of Zhengzhou University, Zhengzhou, Henan, China; ^3^ Surgical Reception Centre, The First Affiliated Hospital of Zhengzhou University, Zhengzhou, Henan, China; ^4^ Department of Urology, Guangzhou Women and Children’s Medical Center, Guangzhou, China; ^5^ Department of Urology, Xinyang Central Hospital, Xinyang, Henan, China; ^6^ Department of Urology, The First Affiliated Hospital of Xinxiang Medical University, Xinxiang, China

**Keywords:** lower urinary tract symptoms, children, adolescents, psychological behavior, risk factors

## Abstract

**Background:**

Lower urinary tract symptoms (LUTS) are clinically frequent and seriously affect the psychological and mental health of children and adolescents. However, most studies on LUTS and its influence on the psychological behavior and mental health have focused on adults. This study aimed to investigate LUTS prevalence and associated factors in children and adolescents and explore its impact on psychological behavior.

**Materials and methods:**

From October 2019 to November 2021, an epidemiological LUTS survey was carried out on 6,077 children aged 6–15 years old in 12 primary and secondary schools in China by using anonymous questionnaires.

**Results:**

A total of 5,500 valid questionnaires were collected, and the total prevalence of four representative symptoms of LUTS: urgency, frequency, daytime urinary incontinence, and nocturnal enuresis was 19.46%, 14.55%, 9.75%, and 8.4%, respectively. The prevalence decreased with age, which decreased rapidly in children aged 6–12 years old. The incidence of LUTS in those who did not continue to use disposable diapers (DD) and began to perform elimination communication (EC) after the age of 1 was significantly higher than that of those who stopped using DD and started EC before 1 year of age (*P* < 0.05). There were significant differences in the occurrence of LUTS without toiled training (TT) (*P* < 0.05). The prevalence of LUTS in males was significantly higher than in females (*P* < 0.05). LUTS in children and adolescents with constipation was significantly higher compared to those without constipation (*P* < 0.05). The detection rate of abnormal psychological behavior in the LUTS group was 44.6%, which was significantly higher than that in the no LUTS group (21.4%, *P* < 0.05). The scores of emotional symptoms, conduct problems, hyperactivity, and peer communication problems were significantly higher in the LUTS group than the control group.

**Conclusions:**

In Mainland China, the prevalence of LUTS in children and adolescents is high. Continued use of DD after 1 year of age, history of urinary tract infection, lack of TT, and constipation were risk factors for LUTS. EC before 1 year of age is a protective factor for LUTS. The prevalence of psychological behavioral abnormalities is high in children and adolescents with LUTS, which needs to be more concerned.

## 1 Introduction

According to the diagnostic criteria of the International Children's Continence Society (ICCS), lower urinary tract symptoms (LUTS) include symptoms in the storage phase, voiding phase, and late voiding phase (1). The main manifestations are urinary urgency, frequency, daytime urinary incontinence (DUI), and nocturnal enuresis (NE). LUTS are poorly understood in children and adolescents due to a lack of medical health education. These issues that seriously affect children and adolescents both physically and psychologically are often neglected by clinicians, resulting in suboptimal clinical care and negative treatment experiences (2, 3). A qualitative exploration of adolescents with incontinence found that young people were despondent and pessimistic about their prognosis, which can influence their coping strategies and adherence to treatment regimes (2).

Interestingly, Grzeda et al. explore whether DUI and NE in children and adolescents are associated with psychosocial problems in adolescence (4). The results found that adolescents with delayed development of bladder control and persistent wetting had increased psychosocial problems in adolescence, further emphasizing the importance of psychological services. Children and adolescents could be at a greater risk of longer-term mental health problems due to peer victimisation, poor self-image, and school experiences (4, 5). Indeed, LUTS in childhood are clinically frequent in clinical practice and risk factors for LUTS can be genetic, demographic, environmental, behavioral, or physical (6). Most studies on LUTS have focused on adults, while fewer studies have examined the prevalence, correlates, and psychological behavior of LUTS in children (7, 8). Additionally, the diagnosis and treatment of LUTS in children and adolescents face numerous challenges (9). Further research on children and adolescents with LUTS is warranted. Therefore, an investigation of associated factors and the impact of LUTS in children and adolescents on psychological behavior can help to understand the current status of LUTS, thereby providing more effective treatment and support for children and adolescents with LUTS. In this study, we investigated the prevalence of LUTS and related factors in 6,077 children and adolescents in China and analyzed its impact on psychological behavior.

## 2 Materials and methods

### 2.1 Study participants

From October 2019 to November 2021, stratified random cluster sampling was used to divide Xinxiang City and Zhengzhou City in Henan Province, China, into three strata: urban, suburban, and township, with two primary schools and secondary schools each randomly selected from each stratum. A total of 6,077 students aged 5–15 years were randomly selected from 12 primary and secondary schools. According to the definition of the ICCS (1), urinary incontinence is defined as persistent or intermittent involuntary loss of urine. DUI is defined as an involuntary leakage of urine during daytime in children 5 years or older. NE is defined as age >5 years and involuntary nocturnal voiding that occurs at least once a month and lasts more than 3 months. Urinary urgency refers to the inability to urinate spontaneously or a feeling of urgency to urinate, which is often difficult to control. Urinary frequency indicates a significant increase in the voiding frequency, i.e., ≥eight times during the day and ≥2 times during the night.

The patients were retained to meet the following criteria: (1) Full-time primary and secondary school students; (2) Children and parents who voluntarily participated in this study; (3) To prevent the influence of relevant drugs on the findings of the survey, requiring that in the last 2 months did not take drugs with the treatment of urological disorders.

Excluded children met the following criteria: (1) Children with other significant comorbidities, such as the functional or anatomical abnormalities of the urinary tract, nervous system, or gastrointestinal tract; (2) Underwent urinary tract or pelvic organ surgery; (3) Diagnosed with LUTS but with other diseases affecting the lower urinary tract or bowel function; (4) Incomplete or inconsistencies in questionnaire responses.

### 2.2 Questionnaire and methods

The questionnaire was composed of three parts (Supplementary File). The first part investigated the demographic characteristics of the children and family such as age, gender, height, caregiver, and lifestyle habits. The second part included urinary urgency, frequency, incontinence, history of urinary tract disorders, history of urinary tract infections (UTIs), defecation and voiding habits, and bowel symptoms. This part was designed based on the generally accepted method of quantitative and standard evaluation of LUTS. The LUTS scoring system, which consists of items regarding daytime symptoms, nighttime symptoms, urinary habits, bowel habits, and quality of life, has been proven as a scientific tool to assess voiding problems in children (10, 11). In this study, the children were divided into two groups according to whether presence of LUTS (including urinary urgency, frequency, incontinence, and UTIs): with no lower urinary tract symptoms group (No LUTS group) and with lower urinary tract symptoms group (LUTS group) (12–14).

The third section was the parent version of the Strengths and Difficulties Questionnaire (SDQ), a short psychological and behavioral screening questionnaire (15). The SDQ comprises 25 items distributed into five scales. Emotional problems, conduct problems, hyperactivity, and peer interaction problems, with higher scores considered abnormal, were added together to obtain a total difficulty score; pro-social behavior, with lower scores considered abnormal. SDQ has been extensively evaluated and widely applied to assess behavior disorders. The reliability and validity of SDQ make it a friendly screening measure of psychosocial problems for children and adolescents (16–18). The threshold values of abnormality for each factor were total difficulty score ≥17, emotional problems ≥5, conduct problems ≥4, peer interaction problems ≥4, hyperactivity ≥7, and pro-social behavior ≤4; the normal level was defined as total difficulty score ≤13, emotional problems ≤3, conduct problems ≤2, hyperactivity ≤5, peer interaction problems ≤2, and pro-social behavior ≥6; the middle range was the borderline status.

In this study, the children and parents completed questionnaires based on the patients' clinical symptoms with the help of a professional medical practitioner. The on-site survey was conducted during parent-teacher conferences, during which the basic concepts and issues of urinary incontinence and NE were explained to parents and children. Questions were answered on-site to maximize the understanding of the survey by children and their parents. After informed consent was given, parents and children were surveyed and filled out the questionnaire together, and all caregivers completed the questionnaire. At the end of the survey, when the questionnaires were collected on the spot, the investigator checked the submitted questionnaires on the spot. For the questionnaires with missing items, the questionnaires were promptly allowed to be filled in by those who filled in the questionnaires to make up for the missing items. However, part of the questionnaires collected during this process were still missing >20% of their contents, which met the exclusion criteria in the present survey study.

### 2.3 Statistical analysis

SPSS 26.0 was used for statistical analysis. Quantitative data was expressed as mean ± standard deviation. Independent samples *T*-test was used for comparison between the two groups. The *χ*² test was used for qualitative data. Logistic regression was used for multiple logistic regression analysis of lower urinary tract symptoms (LUTS). *P* < 0.05 was considered statistically significant.

## 3 Results

### 3.1 Prevalence of LUTS

A total of 6,077 questionnaires were distributed and 5,500 of them (90.51%) qualified for statistical analysis. The number of males and females was 2,879 and 2,621 respectively, with average (10.52 ± 2.87) years old. The distribution of the number of 6–15 years old with increasing age was 542, 552, 531, 566, 540, 552, 560, 558, 556, and 543, respectively.

**Figure 1:**
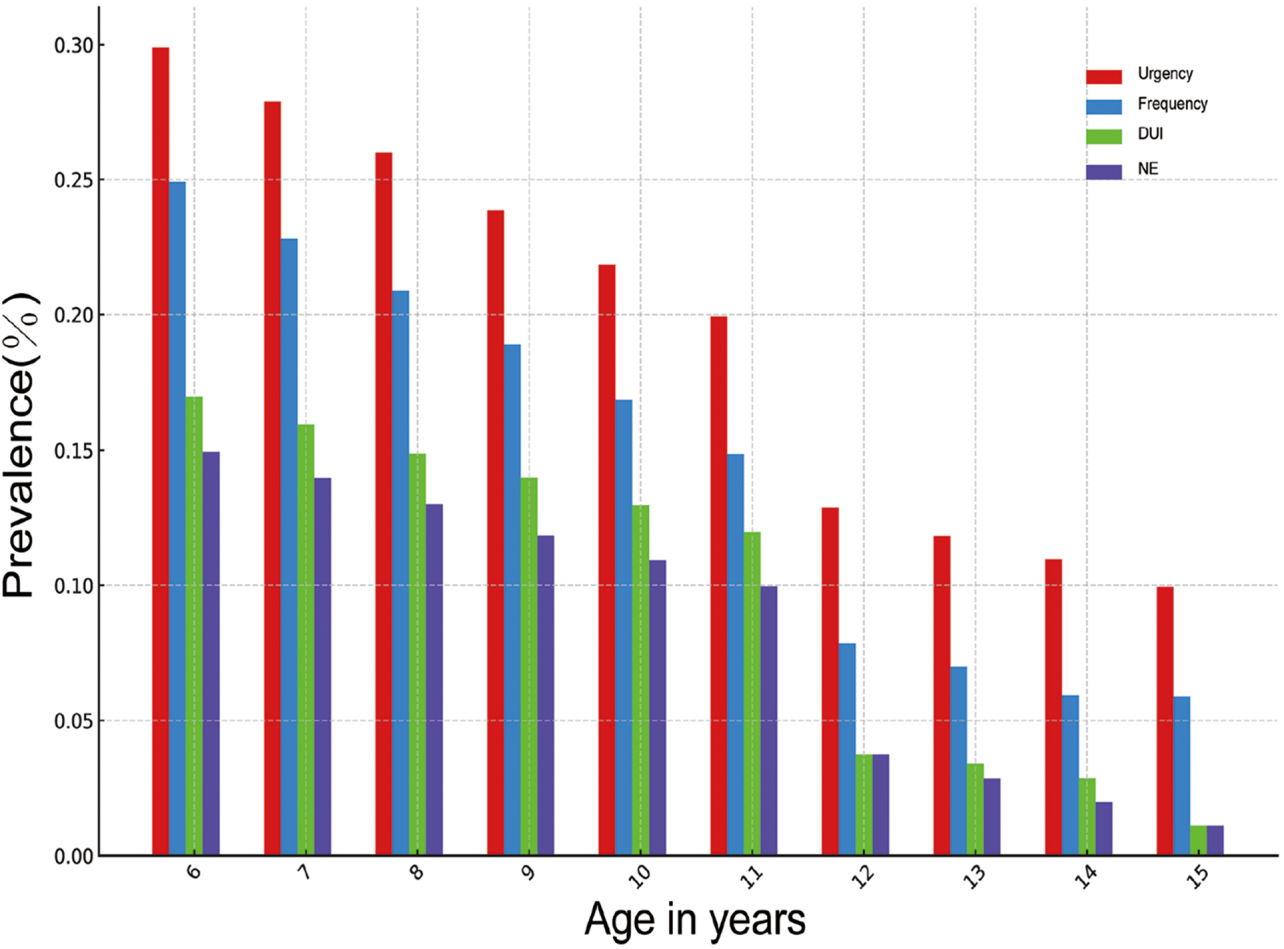
Distribution of the prevalence of LUTS in various age groups. DUI, daytime urinary incontinence; NE, nocturnal enuresis.

The prevalence of LUTS in children of all age groups is shown in Figure 1. The prevalence of LUTS in children shows a gradually decreasing trend with the increase of age, among which the prevalence of LUTS from 6 to 12 years old has a faster-decreasing trend. The prevalence of urinary urgency in children of 6 years old is 29.89%, urinary frequency is 24.91%, NE is 14.94%, and DUI is 16.97%. At the age of 12 years old, the prevalence of urinary urgency decreased to 12.86%, urinary frequency was 7.86%, NE was 3.75%, and DUI was 3.53%. The decreasing trend of each LUTS after 12 years old was significantly slower. The prevalence of urinary urgency by 15 years old decreased to 9.94%, urinary frequency was 5.89%, NE was 1.10%, and DUI was 1.10%. Among children and adolescents aged 6–15 years, there were 1,670 (30.38%) patients with at least one type of LUTS. The highest number was affected by urinary urgency (1,070, 19.46%), and the lowest number was affected by NE (462, 8.40%). Those who suffered from both urinary frequency and urinary urgency accounted for 476 (8.40%), and 250 (4.55%) children and adolescents exhibited all four symptoms.

### 3.2 Risk factor of LUTS

Primary caregiver's education, children's gender, whether or not the use of disposable diaper (DD) after birth lasted until 1 year old, time of initiation of voiding, whether or not children had been toilet-trained, whether or not children had a history of UTIs, whether or not children had been constipated, and whether or not children had a habit of drinking water before bedtime were statistically analyzed. The results showed that boys had a higher prevalence of urinary urgency, urinary frequency, DUI, and NE than girls (Table 1, *P* < 0.001). The incidence of LUTS was higher among children who used DD continuously until more than 1 year of age than among those who did not use disposable diapers or used them less than 1 year of age (Table 1, *p* < 0.001). The incidence of LUTS was lower in children who were elimination communication (EC) before 1 year of age than in those who started after 1 year of age (Table 1, *P* < 0.001). Lack of toilet training (TT) affected the occurrence of urgency, frequency, DUI, and NE (Table 1, *P* < 0.001). The history of UTI and constipation were all associated with the prevalence of urgency, frequency, DUI, and NE (Table 1, *P* < 0.05).

**Table 1:** Statistical analysis of factors influencing the prevalence of urgency, frequency, DUI and NE in children.

Related influencing factors		Urgency			Frequency			DUI			NE		
		*n* (%)	*χ* ^2^	*P*-value	*n* (%)	χ^2^	*P*-value	*n* (%)	χ^2^	*P*-value	*n* (%)	χ^2^	*P*-value
Gender	Girl	402 (15.3%)	54.158	*P* < 0.001	271 (10.3%)	71.259	*P* < 0.001	190 (7.3%)	35.474	*P* < 0.001	153 (5.8%)	42.732	*P* < 0.001
	Boy	668 (23.2%)			529 (18.4%)			346 (12.0%)			309 (10.7%)		
Toilet training (yes/no)	Yes	239 (9.7%)	268.985	*P* < 0.001	172 (7.0%)	203.996	*P* < 0.001	119 (4.8%)	121.704	*P* < 0.001	44 (1.8%)	252.592	*P* < 0.001
	No	831 (27.3%)			628 (20.7%)			417 (13.7%)			418 (13.8%)		
Start EC before one year of age (yes/no)	Yes	275 (11.8%)	153.568	*P* < 0.001	213 (9.1%)	96.649	*P* < 0.001	102 (4.4%)	133.962	*P* < 0.001	52 (2.2%)	201.589	*P* < 0.001
	No	795 (25.1%)			587 (18.6%)			434 (13.7%)			410 (13.0%)		
DD use ≥1 year after birth (yes/no)	Yes	833 (21.1%)	22.818	*P* < 0.001	625 (15.8%)	17.615	*P* < 0.001	437 (11.0%)	26.936	*P* < 0.001	382 (9.7%)	28.73	*P* < 0.001
	No	237 (15.4%)			175 (11.3%)			99 (6.4%)			80 (5.2%)		
History of UTI (yes/no)	Yes	539 (32.7%)	262.584	*P* < 0.001	404 (24.5%)	187.345	*P* < 0.001	293 (17.8%)	172.032	*P* < 0.001	301 (18.2%)	296.767	*P* < 0.001
	No	531 (13.8%)			396 (10.3%)			243 (6.3%)			161 (4.28%)		
Constipation (yes/no)	Yes	440 (25.3%)	55.720	*P* < 0.001	313 (18.0%)	24.526	*P* < 0.001	239 (13.8%)	46.360	*P* < 0.001	212 (12.2%)	47.634	*P* < 0.001
	No	630 (16.8%)			487 (13.0%)			297 (7.9%)			250 (6.7%)		

LUTS, lower urinary tract symptoms; DUI, daytime urinary incontinence; NE, nocturnal enuresis; EC, elimination communication; UTIs, urinary tract infections; DD, disposable diapers.

### 3.3 Multiple logistic regression analysis

The statistically significant factors were included in the multiple logistic regression analysis. Based on Table 2, it is evident that males, using DD at birth >1 year, history of UTIs, and constipation were risk factors for urinary urgency, frequency, DUI, NE, and LUTS (OR >1, *P* < 0.05). Initiation of EC before 1 year old and TT were protective factors for urinary urgency, frequency, DUI, NE, and LUTS (OR <1, *P* < 0.05).

**Table 2:** Results of multiple logistic regression analysis of the prevalence of urgency, frequency, DUI, NE and LUTS in children.

Related influencing factors	Urgency					Frequency				
	B	SE	*P*-value	OR	95%Cl	B	SE	*P*-value	OR	95%Cl
Gender (boy/girl)	0.307	0.075	*P* < 0.01	1.359	1.173–1.574	0.479	0.084	*P* < 0.01	1.615	1.369–1.905
Toilet training (yes/no)	−1.112	0.082	*P* < 0.01	0.329	0.28–0.386	−1.105	0.094	*P* < 0.01	0.331	0.276–0.398
Start EC before one year of age (yes/no)	−0.679	0.081	*P* < 0.01	0.507	0.433–0.594	−0.549	0.09	*P* < 0.01	0.577	0.484–0.688
DD use ≥1 year after birth (yes/no)	0.26	0.089	*P* < 0.01	1.297	1.09–1.543	0.235	0.099	*P* = 0.017	1.265	1.042–1.535
History of UTI (yes/no)	0.913	0.075	*P* < 0.01	2.492	2.149–2.889	0.826	0.083	*P* < 0.01	2.283	1.939–2.688
Constipation (yes/no)	0.319	0.077	*P* < 0.01	1.376	1.182–1.601	0.187	0.086	*P* < 0.01	1.205	1.018–1.426
Related influencing factors	DUI					NE				
	B	SE	*P*-value	OR	95%Cl	B	SE	*P*-value	OR	95%Cl
Gender (boy/girl)	0.287	0.1	*P* < 0.01	1.332	1.095–1.621	0.224	0.113	*P* = 0.047	1.251	1.003–1.56
Toilet training (yes/no)	−0.934	0.111	*P* < 0.01	0.393	0.316–0.489	−1.971	0.165	*P* < 0.01	0.139	0.101–0.192
Start EC before one year of age (yes/no)	−0.98	0.118	*P* < 0.01	0.375	0.298–0.473	−1.563	0.156	*P* < 0.01	0.209	0.154–0.284
DD use ≥1 year after birth (yes/no)	0.477	0.124	*P* < 0.01	1.612	1.265–2.055	0.478	0.14	*P* < 0.01	1.613	1.227–2.122
History of UTI (yes/no)	0.879	0.098	*P* < 0.01	2.409	1.987–2.92	1.345	0.111	*P* < 0.01	3.837	3.089–4.766
Constipation (yes/no)	0.425	0.099	*P* < 0.01	1.53	1.26–1.858	0.345	0.11	*P* < 0.01	1.412	1.139–1.751
Related influencing factors	LUTS									
	B		SE		*P*-value		OR		95%Cl	
Gender (boy/girl)	0.438		0.068		*P* < 0.01		1.55		1.357–1.77	
Toilet training (yes/no)	−1.454		0.072		*P* < 0.01		0.234		0.203–0.269	
Start EC before one year of age (yes/no)	−1.286		0.073		*P* < 0.01		0.276		0.239–0.319	
DD use ≥1 year after birth (yes/no)	0.772		0.081		*P* < 0.01		2.163		1.844–2.538	
History of UTI (yes/no)	0.313		0.071		*P* < 0.01		1.367		1.189–1.572	
Constipation (yes/no)	0.589		0.071		*P* < 0.01		1.802		1.567–2.072	

LUTS, lower urinary tract symptoms; DUI, daytime urinary incontinence; NE, nocturnal enuresis; EC, elimination communication; UTIs, urinary tract infections; OR, odds ratio; DD, disposable diaper.

### 3.4 Psychological behavioral problems

The detection rate of clinically relevant psychological or behavioral symptoms in the LUTS group was 44.6% (745/1,670), which was significantly higher than that of the No LUTS group (821/3,830, 21.4%), with a statistically significant difference (*p* < 0.05). Emotional symptoms, conduct problems, hyperactivity, peer interaction problems, and total SDQ scores of the LUTS group were higher than those of the control group (*p* < 0.05). The difference in pro-social behavior scores between the two groups was insignificant (Table 3).

**Table 3:** Comparison of psychological and behavioral problems between the LUTS group and the normal group.

SDQ scale	LUTS (*n* = 1,670)	No LUTS (*n* = 3,830)	*T*-value	*P*-value
Emotional symptoms	4.24 ± 2.67	2.86 ± 2.03	21.37	*P* < 0.001
Conduct problems	3.70 ± 2.17	2.65 ± 1.26	22.18	*P* < 0.001
Hyperactivity	4.37 ± 2.23	3.74 ± 1.97	10.39	*P* < 0.001
Peer interaction	3.94 ± 2.45	3.22 ± 1.35	13.95	*P* < 0.001
Pro-social behavior	5.93 ± 1.42	5.99 ± 1.18	1.43	*P* = 0.097
Total SDQ scores	22.21 ± 8.53	18.54 ± 5.66	18.54	*P* < 0.001

LUTS, lower urinary tract symptoms; SDQ, strength and difficulties questionnaire.

## 4 Discussion

LUTS are a syndrome of nonspecific urinary symptoms that include storage, voiding, and postvoiding discomfort. Epidemiologic studies have shown rates of incontinence and NE as high as 20% in school-age children. Vaz et al. (19) reported a prevalence of 21.8% for LUTS in Brazilian school-age children, suggesting that LUTS must be investigated carefully at routine pediatric visits. However, there is a lack of data on the prevalence of LUTS in Chinese children. Previous research indicated that factors such as the use of DD after birth, TT, and EC during infancy can influence subsequent voiding control (20, 21). Simultaneously, with improvements in lifestyles and changes in parenting practices, there is a rising trend in the prevalence of LUTS in Chinese children and adolescents, with a significant increase in the overall prevalence of primary enuresis in 2017 compared to 2006 (7.30% vs. 4.07%) (22, 23). Another explanation for the “rising trend” in the prevalence of LUTS could be that more medical education led to an increased focus on LUTS, more advanced and varied screening methods for the detection of LUTS, and more information about these diseases, which have led to more children being diagnosed and treated. In this study, NE showed an increased risk in 6–15 years old. The results also indicated a higher prevalence of LUTS in 6–15 years old, with a total of 1,670 patients (30.38%) with at least one type of LUTS. Nevertheless, there is a gradual decrease in the prevalence of all four kinds of LUTS with age.

The use of diaper-related products has alleviated parental anxieties/burdens and provided convenience. However, over the past few decades, the prolonged and extensive reliance on DD has become a significant factor contributing to the increased prevalence of NE (24). Through epidemiological investigations, this study confirmed that the use of DD beyond the 1 year old has a significant impact on four representative symptoms of LUTS (*P* < 0.05). Therefore, the voiding abnormalities associated with the use of DD constitute a noteworthy concern for parents. EC is known as “natural infant hygiene” and is sometimes referred to as “baby-led potty training” or “assisted infant toilet training”. Elimination refers to the act of defecation or urination. Elimination communication is a two-way process in toilet training. When the child shows cues of elimination, such as crying, squirming, straining, wriggling, grimacing, fussing, and vocalizing, the caregiver can coordinate this elimination process with audio cues (soft whistle or hum) whilst holding or sitting the child with thighs apart over the toilet to complete this process rather than to allow them to eliminate in their DD (20, 21). There is still controversy surrounding the effect of disposable diaper use on continence. In mainland China, the incidence of LUTS was higher among children who used DD continuously until more than 1 year of age. Other studies also showed that increased age of daytime DD use for children aged 5 years had a positive correlation with NE prevalence (22, 24, 25). Nevertheless, there is evidence from other countries where diapers are used for much longer periods (up to the age of 2 or 3), and where LUTS are not more common than in China (26, 27). The study by Duong et al. found that children who did not wear DD had earlier continence (28). Therefore, the effect of DD on LUTS still requires further investigation. It is evident that multicenter prospective studies are needed in the future to provide more definitive conclusions. The study also revealed that EC before the age of 1 has a significant mitigating effect on LUTS in children (*P* < 0.05). EC before the age of 1 is beneficial for urinary control in early childhood, thereby reducing the occurrence of LUTS in children and adolescents (24). Vermandel et al. (29) defined TT as the conscious process in which caregivers bring the child to the toilet and guide them to use it, with a frequency of no less than 1–2 times per day. They proposed that TT contributed to better bladder control in children, consequently reducing the prevalence of LUTS (30, 31). Our results indicated that children who underwent TT were effectively able to reduce the prevalence of the four types of LUTS. Therefore, using DD before the 1 year old, EC before the 1 year old, and actively conducting TT are beneficial for voiding control in children, which helps reduce the occurrence of LUTS.

Noteworthy, it has been demonstrated that defecation problems and LUTS could occur together (32, 33). In this study, children with constipation were found to be more prone to LUTS. Averbeck et al. suggested that the bladder and rectum, having a common embryonic origin and similar innervation, may mutually interfere with each other. When a child is constipated, this can lead to the bladder's inability to store and empty completely, causing concurrent issues with voiding and defecation (32). Therefore, there is a need to pay attention to functional impairments in both the intestinal and bladder systems in children, which can impact overall treatment outcomes.

LUTS not only affects the mental health of children and adolescents, but may also trigger or exacerbate psychological problems such as anxiety, low self-esteem, and social difficulties (34, 35). Children with LUTS might avoid participating in social and psychological activities due to embarrassment or fear of ridicule, which can adversely affect their social skills and overall well-being. Numerous studies have found that DUI and NE cause psycho-behavioral problems in children (36, 37). Moreover, children suffering from incontinence were more likely to present with neurodevelopmental disorders such as attention-deficit/hyperactivity disorder, autism spectrum disorder, and intellectual disability, which further led to children's peer interaction disorders and family communication disorders (38). Our study found that emotional and behavioral problems were detected at a rate of 44.6% (745/1,670), which was significantly higher than the normal group of children. Therefore, it is essential to analyze psycho-behavioral factors affecting children with LUTS, thereby giving appropriate guidance accordingly and diverting the patient's emotional distress. Counseling with a professional psychologist is warranted if the children suffer from more serious psychological or psychiatric problems. These strategies can promote the treatment of LUTS in children (39).

Our investigation delves into the LUTS prevalence and associated factors in children and adolescents and explores its impact on psychological behavior. Notably, our research endeavors to bridge a gap in existing clinical psychological literature concerning Chinese children with LUTS by highlighting the significance of psycho-behavioral interventions. However, some limitations should be acknowledged. The sample size is derived from a specific region in China, which may not fully represent children throughout China. Additionally, the anonymous survey relies on retrospective data, particularly in recalling precise details such as the initiation and conclusion of TT. These limitations highlight the need for more in-depth research in the future.

## 5 Conclusion

In Mainland China, the prevalence of LUTS in children and adolescents is high, and psycho-behavioral abnormalities are more prevalent in children with LUTS. Risk factors for LUTS include the continued use of DD after 1 year old, a history of UTIs, lack of TT, and constipation. Conversely, EC before 1 year old is identified as a protective strategy. Notably, children with LUTS have severe emotional and behavioral disturbances, leading to reduced treatment adherence. Psychological counseling and health education for children with LUTS should be strengthened to ensure optimal care and delivery of the most favorable results.

## Data Availability

The original contributions presented in the study are included in the article/Supplementary Material, further inquiries can be directed to the corresponding author.
