# Supplementary material for: Prevalence, risk factors, psychological effects of children and adolescents with lower urinary tract symptoms: a large population-based study
Source: Front Pediatr. 2024 Aug 21;12:1455171. doi: 10.3389/fped.2024.1455171 (PMC11371695; doi:10.3389/fped.2024.1455171)
Supplement: Supplementary file 1 [file Datasheet1.pdf]

**Investigation on the incidence of prevalence, risk factors, psychological effects of children and adolescents with lower urinary tract symptoms**

Dear parents and children,

Thank you for completing the questionnaire on the incidence of Lower urinary tract symptoms (urgency, frequency, daytime urinary incontinence (DUI), and nocturnal enuresis (NE)) and the factors affecting it in elementary and junior high school students. The purpose of this survey is to investigate the incidence of prevalence, risk factors, psychological effects of children and adolescents with lower urinary tract symptoms and to provide reference for the prevention and treatment of lower urinary tract symptoms in adolescents. This survey is anonymous, there is no leakage problem, the questionnaire is filled out and put directly into the questionnaire collection box. In addition, you can also participate in the online version of the survey, fill out the web form and submit it. Don't pay attention to the advertisements that appear after submitting the survey form. It would be appreciated if you can help WeChat Survey and forward the questionnaire in your circle of friends! Scan the QR code below to start participating in the survey.

****In order to complete the questionnaire correctly, please read the following concepts before you begin to complete the questionnaire.**

Urinary frequency: refers to urinating more than 8 times per 24 hours.

Urinary urgency: refers to the inability to self-control urination or urination with a sense of urgency, without timely urination, then urinary incontinence will occur.

Urge incontinence: refers to when there is a strong urge to urinate can not be controlled by the will and urine out through the urethra.

Stress incontinence: refers to involuntary overflow of urine when there is an increase in abdominal pressure such as coughing, sneezing and laughing.

Urinary leakage: This is the unconscious leakage of urine during sleep, commonly known as “bedwetting”. It occurs at least once every three months.

Nocturia: refers to getting up to urinate during sleep and then going back to sleep.

Hypospadias: refers to the foreskin normally covering the urethral opening, but not

being able to be turned out to expose the urethral opening and the head of the penis.

Concealed Penis: means the body of the penis is shrunken and hidden inside the skin, and the only thing that protrudes outside is the foreskin. If the skin of the penis is squeezed inward with the hand, the body of the penis will be revealed, and when the hand is slightly released, the body of the penis will retract.

Penile scrotal transposition: This is when the scrotum is situated abnormally above the penis.

Cryptorchidism: refers to the testicles not being in the scrotum.

If you have any health problems, you can contact Dr. Jianguo Wen (phone and WeChat number +8613939068686) for consultation. Prof. Jianguo Wen (Dual M.D., Ph.D. Supervisor, Member of the Urodynamics Committee of the International Council of Urological Controls (ICS) and Principal of the School of Adolescent Urology: https://www.ics.org/institute/paediatric)

**The first part: Demographic characteristics of the children and family (Please tick (√) or fill in the blanks for the following questions)**

1. Sex of child: ①Male□ ②Female□; Date of birth: _

Place of birth: County □ Rural □; if the place of birth is rural, the child moved to the county at the age of _ to attend school and live there. Current Height: _centimeters; Weight: _kilograms

2. Child's primary caregiver (multiple choice): ①parents□ ②grandparents□ ③nanny□

3. Occupation of the main caregiver: ①worker□; ②farmer□; ③administration□; ④commercial□; ⑤military□; ⑥kindergarten teacher□, other occupations:_. Educational level: ①High school and below□ ②College and bachelor degree or above□

4. How is your child's academic performance? ①Excellent□ ②Good□ ③Moderate□ ④Passing□ ⑤Failing□

5. What kind of personality does your child have (Multiple choice) ①Outgoing □

②Introvert □ ③Solitary □ ④Intemperate □

6. Your child usually defecates _ times during the day (from the time he/she wakes up in the morning to the time he/she goes to bed at night), and _ times during the night (from the time he/she falls asleep to the time he/she wakes up the next day)

7. Your child usually urinates _ times during the day (from the time he/she wakes up in the morning to the time he/she goes to bed at night, including the first time he/she wakes up in the morning), and _ times during the night (from the time he/she falls asleep to the time he/she wakes up the next day, excluding the first time he/she wakes up in the morning)

8. Have you used diapers since your child was born? ① use □; ② never used □

9. If you use diapers, answer the following questions: (If not, please skip to question 15)

①used mainly at night, ending at □□□ years old □□□ months; ②used both at night and during the day, ending at □□□ years old □□□ months

10. If your child has been using diapers since birth, on average, he/she uses _ diaper per day.

11. Did you experience any of the following when using diapers: prickly heat□red and itchy skin□other symptoms_?

12. Is there any long-term dependence on the use of diapers? (without the use of diapers, the child will be crying, incontinence, etc.) ①yes □; ② no □

13. Have you ever trained your child to urinate and defecate? ① Never trained □; ② occasionally trained □; ③ often trained □ (regular □, irregular □)

14. When did your child start to urinate? If urinating ① □ times during the day; ② □ times during the night;

15. Child's bowel training began when he/she was □□□□ years old and □□□□ months old. If you use the toilet, □ times a day;

16. Do you think that the earlier the child is trained to “pee and poop”, the better the child's ability to control urine and stool will be? ①Agree □ ②Disagree ③Don't know

17. Is there any immediate family member over 5 years old who still wet the bed:

Yes□; No□; If yes (please check) which relative Father□Mother□Grandfather□Mother□Grandmother□Grandfather□Brother□Brother□ Sister□Sister□Mother

18. Is your child easily awakened during sleep: □ Not easily awakened □ Easily awakened

19. Does your child have the habit of drinking or eating before going to bed (within 2h)? Yes□; No□

**The second part: Scale of lower urinary tract symptoms in children (LUTSS)**

**(For the tables below, please check the appropriate box. Please base your answers on this child's lower urinary tract symptoms in the past six months or during the school year. Be sure to answer each question, even if you are not quite sure about a particular question.)**

| | | | | |
|---|---|---|---|---|
| 1. Does your child wet during the day? | No | Sometimes | 1-2 times / day | Always |
| 2. How wet is your child during the day? | Damp underwear | Damp pants only | Pants soaking wet | |
| 3. Does your child wet during the night? | No | 1–2 Nights/ week | 3–5 Nights/ week | 6–7 Nights/ week |
| 4. How wet is your child during the night? | Damp underwear | Damp bed sheet only | Bed sheets soaking wet | |

| | | |
|---|---|---|
| | | |
| 5. How many times does your child void? | ＜1－7/Day | More than7/day |
| 6. My child is too quick to finish his/her pee. | No | Yes |
| 7. My child strains during voiding. | No | Yes |
| 8. My child feels pain during voiding. | No | Yes |
| 9. My child voids intermittently. | No | Yes |
| 10. My child needs to go back voiding soon after finishes his/her pee. | No | Yes |
| 11. My child has a sudden feeling of having to urinate immediately. | No | Yes |

| | | | | |
|---|---|---|---|---|
| 12. My child holds by crossing his/her legs. | No | Yes | | |
| 13. My child wets on the way to the toilet. | No | Yes | | |
| 14. My child misses his/her bowel movement every day! | No | Yes | | |
| 15. Does your child use diapers? | No | Yes | | |
| 16. If your child has used diapers, how often were they used? | daily | several times a week | occasionally | |
| 17. What time of day do you usually diaper your child? | day | night | all day | |
| 18. What is the age your child stopped using diapers? | <1 years old | ≥1 years old and <3 years old | ≥3 years old | |
| 19. Do you implement toilet training for your children? | No | Yes | | |
| 20. Is there a delay in starting toilet training? | No | Yes | | |
| 21. What is the age your child started toilet training? | <1 years old | ≥1 years old and <3 years old | ≥3 years old | |
| Quality of life | | | | |
| If your child experiences symptoms mentioned above, does it affect his/her family, social or school life? | No | Sometimes | Yes affects | Seriously affects |

**The third part: The parent version of the Strengths and Difficulties Questionnaire (SDQ) (For the tables below, please check the appropriate box. Please base your answers on this child's psychological and behavioral in the past six months or during the school year. Be sure to answer each question, even if you are not quite sure about a particular question.)**

| | Yes | Medium | No |
|---|---|---|---|
| Be able to sympathize with the feelings of others | | | |
| Unstable, overactive, unable to meditate for long periods of time | | | |
| Often complains of headaches, stomach aches, or nausea | | | |
| Be happy to share things with other children (candy, toys, pens, etc.) | | | |
| Frequent temper tantrums and is easily angered | | | |
| Quite solitary, more likely to play by themselves | | | |
| Generally submissive, usually doing whatever adults ask them to do | | | |
| Worries a lot and shows worry a lot | | | |
| Willing to help if someone is hurt, upset or sick | | | |
| Will continually fidget or wiggle their arms and legs when seated | | | |
| At least one good friend | | | |
| Often fights with or bullies other children | | | |
| Be often upset, moody or crying | | | |
| Be generally liked by other children | | | |

| | | | |
|---|---|---|---|
| Easily distracted and unable to give full attention | | | |
| Be nervous or clingy in new situations and lose confidence easily | | | |
| Be kind to younger children | | | |
| Lies or cheats a lot | | | |
| Be teased or bullied by other children | | | |
| Often volunteers to help others (parents, teachers, or other children) | | | |
| Thinks before doing things | | | |
| Stealing things from home, school, or other places | | | |
| Gets along better with adults than children | | | |
| Be afraid of many things and gets frightened easily | | | |
| Follows through on things and has a long attention span | | | |

| Emotional problem<br><br>If more than two items are positive: a full screening is required | | |
|---|---|---|
| 1. does your child sometimes feel that others react negatively? | Yes | No |
| 2. Does your child sometimes feel worthless or lack self-confidence? | Yes | No |
| 3. Does your child sometimes get headaches? | Yes | No |
| 4. Does your child sometimes feel sick? | Yes | No |
| 5. Does your child sometimes have abdominal pain? | Yes | No |
| 6. Does your child sometimes lack energy? | Yes | No |

7. Does your child sometimes feel unhappy, sad or depressed? Yes No

Symptoms of Inattention

If more than two items are positive: full screening is needed

1. Does your child often fail to pay attention to details or do careless homework? Yes No

2. Does your child often have difficulty organizing tasks and activities? Yes No

3. Does your child often forget during daily exercises? Yes No

Hyperactivity/Impulsivity Symptoms

Your child ......

4. Does your child often talk continuously? Yes No

5. Is your child often busy? Yes No

6. Does your child often run or climb in inappropriate situations? Yes No

| | Yes | No | Don't know |
|---|---|---|---|
| Does your child do any of the following during sleep ...... | | | |
| Snores for more than half of the sleep time | | | |
| Snores all the time | | | |
| Snores loudly | | | |
| Has “heavy” or loud breathing? | | | |
| Difficult or labored breathing? | | | |
| Have you ever found ...... | | | |
| Does your child have apnea at night? | | | |
| If more than two positive items: full screening is required | | | |

| | | | |
|---|---|---|---|
| Tend to breathe through the mouth during the day? | | | |
| Does he or she wake up in the morning with a dry mouth? | | | |
| Wet the bed occasionally? | | | |
| Wake up feeling sick in the morning? | | | |
| Any sleepiness during the day? | | | |
| Has a teacher or other guardian responded that your child is sleepy during the day? | | | |
| Does your child wake up easily in the morning? | | | |
| Does your child wake up with a headache in the morning? | | | |
| Has your child had a period of growth retardation since birth? | | | |
| Is your child overweight? | | | |
| Does your child often ...... | | | |
| seems to be listening (not paying attention when listening to what is being said) | | | |
| Has difficulty being set up or organized | | | |
| Is easily distracted by external stimuli | | | |
| Fidgets or sits unsteadily on hands or feet | | | |
| Walks around, is busy or boastful | | | |
| Interrupts or disturbs others (e.g., when playing games) | | | |
